# Puerperal Convulsions—Induced Labor—Death of the Mother

**Published:** 1881-08

**Authors:** H. R. Hopkins


### Clinical Reports.

### Puerperal Convulsions—Induced Labor—Death of the Mother.

Reported by H. R. Hopkins, M. D.

I was called to see Mrs. O., at one o'clock in the morning of the 18th ult. On reaching the house I found the woman lying in complete insensibility, and heard the following history: The couple married but about a year since, and had recently come to live in the city. The wife, a perfectly healthy young woman of 32 years, was the youngest daughter of a large family of sisters, several of whom had families of from three to five children. Finding herself pregnant she had not thought it necessary to take professional advice for herself, but conferred with her sisters freely and was assured by them that everything was progressing in a perfectly normal manner. Her husband said that she had in general been in perfect health, but had complained of a very unusual pain in the head during the afternoon and evening of the day before my visit. That for a month her feet had swelled some, and during the last week she had also complained of swelling in her hands, but that they thought nothing of it, but supposed it was usual with women in her condition. He remembered that she complained some of her head after they got to bed, but thought that she was asleep in a reasonable time; but that she awakened him soon after midnight by her peculiar movements, which he found was a convulsion. The lady who stayed with her while the husband came for me, said that she was unconscious during the whole time of his absence.

She was profoundly unconscious, breathing heavily, almost stertorously, with dilated pupils, slightly bloody froth on the lips, a hot, dry skin, swollen limbs and extremities, and a hard, long, slow pulse of 40 to 44 per minute.

After sending for assistance I gave her chloroform for a few moments and the pulse rose to 60, but did not soften. I then took two-thirds of a pint of blood from the arm with effect of raising the pulse to 96 and with a complete restoration to consciousness. She was quite surprised to find what had happened; did not know that she had been ill in any way, and said she did not expect to have her baby for six weeks at least. External palpation and digital examination showed no signs of labor. Dr. Boardman agreed with me that she should be put at once under the influence of bromide of potassium and chloral, with chloroform upon signs of returning convulsions, and after giving the nurse such instructions and the husband a very guarded prognosis, we left the house, I taking with me a few drams of urine drawn by the catheter. The prognosis was not improved upon seeing this turn completely solid upon adding a few drops of nitric acid. Was called again at 7 A. M., and found that she had remained comfortable until about 6.30, when she had a slight convulsion, and that since then she had been unconscious. At 7.30 had a slight attack. I thought cut short by the chloroform. Has taken four grams each of bromide of potash and chloral by the mouth, and is to have two grams of each every hour per rectum as needed. Could see no signs of uterine contraction. She was free from convulsions until 10 A. M., when she had an attack of extreme violence, followed by another in half an hour. At noon there were slight signs of uterine contractions, the os admitting the finger easily. At 4 P. M. found that she had had no convulsions since 10.30, but was still unconscious. The pulse had gradually risen during the forenoon, was noted at 96, then 100, then 110, and at this time was fully 120 and irregular in both force and time. Dr. Boardman agreed with me that delivery must be induced at once, if we would save either of the lives now in imminent peril.

The uterus could be felt contracting and the os would admit two finger tips. Dr. Boardman began the dilatation and in 15 minutes introduced the ends of the thumb and four fingers. Becoming exhausted, I relieved him and soon passed my hand into the uterus. I then closed my hand and partly withdrew and partly allowed the uterus to expel the fist through the now yielding os. The head followed immediately, was grasped by the forceps, and at a quarter to 5 delivered a living male child of about eight pounds weight. The uterus contracted promptly, the secundines were removed, the os was felt to be free from rupture, and there was no hemorrhage. In spite of our best efforts at supporting and stimulating, the mother showed no signs of returning consciousness and died at ten the following forenoon, about 24 hours from the time of her last convulsion. The child seemed rather feeble at first, but soon rallied and has thrived continuously. This case seems to me, perhaps, worthy of record for various reasons.

It is a most striking illustration of one of the dangers of child-bearing. The patient was intelligent, and possessed of abundant means, and yet she allowed disease to slowly undermine the foundations of health and life, without even asking for advice. An examination of her urine would probably have shown the kidney disease to have been of considerable duration, and certainly would have indicated treatment with a fair prospect of saving her life.

I was perfectly clear as to the propriety of the blood-letting, and the return of consciousness would seem to vindicate the value of the measure; at the same time the rise in the pulse during the morning was taken by Dr. Boardman and myself as a warning that further treatment in that direction would not be justifiable. From first to last the circulatory phenomena were most interesting; the long, hard, slow pulse of the first visit was most peculiar, and the gradual early heart failure prominently marked. At 4 P. M. the pulse was 120, and irregular; at 6 P. M. it was 140, at 8 P. M. I could not count it, and at 10 P. M. could not feel it, nor did I again find the pulsation, although the breathing went on for twelve hours. The readiness with which the os yielded to the dilating force of the hand, and the short time required for delivery, are items of favorable testimony for this means of hastening labor in cases of great emergency.

